# Editorial: The Least Cost Path From Landscape Genetics to Landscape Genomics: Challenges and Opportunities to Explore NGS Data in a Spatially Explicit Context

**DOI:** 10.3389/fgene.2018.00215

**Published:** 2018-06-19

**Authors:** Samuel A. Cushman, Andrew J. Shirk, Glenn T. Howe, Melanie A. Murphy, Rodney J. Dyer, Stéphane Joost

**Affiliations:** ^1^USDA Forest Service, Rocky Mountain Research Station, Flagstaff, AZ, United States; ^2^Climate Impacts Group, University of Washington, Seattle, WA, United States; ^3^Forest Ecosystems and Society, Oregon State University, Corvallis, OR, United States; ^4^Ecosystem Science and Management, University of Wyoming, Laramie, WY, United States; ^5^Center for Environmental Science, Virginia Commonwealth University, Richmond, VA, United States; ^6^Laboratory of Geographic Information Systems (LASIG), School of Architecture, Civil and Environmental Engineering (ENAC), École Polytechnique Fédérale de Lausanne (EPFL), Lausanne, Switzerland

**Keywords:** landscape genetics, landscape genomics, next generation sequencing, spatial genetics, ecological genetics

Ecosystems are the stage on which the play of evolution is acted. Inferring evolutionary processes from the spatial and temporal genetic patterns they produce in populations is challenging because ecosystems are highly complex, spatially structured, and temporally varying. The field of landscape genetics has offered a means of navigating these challenges to make eco-evolutionary insights for many species. The emerging field of landscape genomics offers great promise to expand the potential of landscape genetic analysis even further. The purpose of this Research Topic for Evolutionary and Population Genetics is to explore a number of critical challenges and opportunities for the transition from landscape genetics to landscape genomics. To-date, landscape genetics has generally focused on spatial analyses of small genetic datasets, typically comprised of <20 microsatellite markers, taken from clusters of individuals in putative “populations” or distributed individuals across landscapes. The recent emergence of large-scale genomic datasets containing thousands of markers produced by next generation sequencing (NGS) methods poses tremendous opportunity and challenge to the field. Perhaps the greatest is to produce, process, curate, archive, and analyze spatially referenced genomic datasets in a way such that research is led by a priori hypotheses about how environmental heterogeneity and temporal dynamics interact to influence gene flow and selection. Effective progress in this transition to a robust field of landscape genomics will likely depend on integrating vast genomic datasets with powerful modeling and replicated and controlled experiments to test putative relationships between population processes and evolutionary and population genetic responses (Cushman, 2014). The recent availability of whole genome sequence (WGS) data offers incredible molecular resolution, but comes at great expense. This limits the spatial and temporal sample sizes for economic reasons, making it challenging to achieve spatial representativeness and temporal robustness.

No single person has the expertise or the time to effectively bring these components together. More than ever, success in advancing our field will depend on collaborations across large multi-disciplinary groups (Cushman, 2014). Experts in the development of genomic, epigenomic, and transcriptomic data from high throughput technologies are needed to produce the genome-wide raw data for subsequent analysis. Bioinformatics specialists are needed to provide programming and computer science expertise to efficiently handle and analyze vast genomic datasets, and to effectively utilize high performance computing resources. Modelers will be needed to work with the bioinformaticians to explore the implications of hypotheses a priori, to refine hypotheses by optimizing fit to observed data, and predict how observed pattern-process relationships may propagate across scale through space and time. Experimenters should work closely with modelers to rigorously test hypotheses in controlled and replicated experiments. To be successful, this entire integration should be led by theoreticians who have a coherent vision for how each of these parts will synergize to address focused and falsifiable questions of importance in advancing the field.

In this research topic we recruited a number of leading experts in genomics, epigenetics, landscape genetics, and simulation modeling to explore the challenges and opportunities presented by the intersection of NGS data, spatial modeling, and replicated and controlled experimentation. Overall, this effort produced a series of 10 papers, not including this editorial. These papers covered a wide range of topics including (1) two reviews of recent developments and current status of landscape genomics, (2) one review of theory and mechanisms of epigenetics and their applications in a landscape genomic context, (3) two papers illustrating the cutting edge in individual-based, spatially-explicit simulation modeling applied to eco-evolutionary problems in landscape genomics, (4) one paper about using genetic rare variants, or singletons, to infer past demographic events over a species' history, (5) three empirical papers describing a range of analytical methods to explore the spatial and environmental drivers of selection and genetic differentiation in plants and animals, and (6) one paper focused on the landscape side of “landscape genomics” which provides a review and evaluation of best practices of using geographical information systems to compile, display and analyze environmental data in the most appropriate way for landscape genomic research.

Landscape genomics is at the exciting cutting edge of the recent spatial revolution that has led to the emergence of the field of landscape genetics. Given the recency of landscape genomics as a field of study, there are relatively few established research frameworks, analytical approaches or even conceptual models for what is meant by landscape genomics and how it is best conducted (see Balkenhol et al., 2017). The two reviews of recent landscape genomics literature in this Research Topic attempt to summarize the field as it stands now and identify its strengths, weaknesses and opportunities. In the first of these two review papers, Li et al. define landscape genomics as a new discipline that aims to reveal relationships between adaptive genetic variation and environmental heterogeneity, and note that there have been few formal landscape genomics papers published to date. Their review outlines the sampling strategies, molecular marker types and research categories in 37 articles published during the first 10 years of this field, and identifies major challenges and future directions for landscape genomics. The second review, by Storfer et al., emphasizes the role of emerging genomic technology in driving the emergence of landscape genomics as a field of study. In particular, they note that widely available next-generation sequencing data have resulted in immensely improved ability to detect candidate genes under selection and identify the environmental factors that drive that selection. However, they note that the transition between landscape genetics and landscape genomics is extremely challenging due to the difficulty of handling and interpreting vast genomic datasets. They also note the rapid emergence of a wide range of analysis methods and provide detailed discussion of outlier differentiation methods and genetic-environment association tests. They note that the key to choosing appropriate genome scan methods is an understanding of the underlying demographic structure of study populations, and such data can be obtained using neutral loci from the generated genome-wide data or prior knowledge of a species' phylogeographic history and summarize recent simulation studies that test the power and accuracy of genome scan methods under a variety of demographic scenarios and sampling designs. They conclude with a discussion of additional considerations for future method development, and a summary of methods that show promise for landscape genomics studies but are not yet widely used. These two reviews provide what is probably the most complete snap-shot of the field of landscape genomics produced to-date, and propose an excellent foundation for the more theoretical papers in the Research Topic as well as context for the papers that present empirical examples of current landscape genomic research.

Epigenetics has recently emerged as a topic of immense interest in evolutionary biology. Up to this time, landscape genetics and landscape genomics research has focused on sequence genetic variation in relation to natural gene flow and adaptive variation. However, it is appearing increasingly likely that a large portion of the variance in evolutionary responses is related not to variation in genomic sequences but to epigenetic regulation of the expression of those sequences. Fitness-related traits can be affected by heritable variation in epigenetic marks, resulting in transgenerational plasticity. Given the importance of epigenetics in evolutionary biology, it is critical to begin the integration of epigenetics with landscape genetics and landscape genomics (e.g., Paun et al., 2010). Whipple and Holeski take an exciting first step in this effort with their review of epigenetic theory and mechanisms and their relationships with landscape genomics and landscape genetics. In their paper they summarize the relevance of epigenetic inheritance to ecological and evolutionary processes, and review the literature on landscape-level patterns of epigenetic variation. They argue that landscape-level patterns of epigenomic variation in plants generally show greater levels of isolation by distance and isolation by environment than is found for the genome, suggesting a perhaps elevated role in the spatial population processes that are the focus of landscape genetics and genomics. They note that demonstrating transgenerational inheritance requires more complex breeding and/or experimental designs, and argue that multi-generation common garden experiments conducted across multiple environments are required to understand epigenome inheritance and to separate the relative contributions of heritable epigenetic variation to the phenotype.

The two papers in the Research Topic that focus on individual-based, spatially-explicit simulation modeling of eco-evolutionary processes offer a tantalizing glimpse into the exciting emerging field of landscape genomic simulation modeling. In the first of these papers, Landguth et al. present the first application to a real-world ecological system of a new individual-based simulation model that incorporates spatially complex gene flow and spatially heterogeneous environmentally driven selection. They use the recent population declines to the high elevation western North America foundation species whitebark pine as a case study to illustrate the power of this modeling framework. Specifically, they present a simulation modeling framework to improve understanding of the long-term genetic consequences of the blister rust pathogen, the evolution of rust resistance, and scenarios of planting rust resistant genotypes of whitebark pine. By combining climatic niche modeling and eco-evolutionary landscape genetics modeling, they evaluate the effects of different scenarios of planting rust-resistant genotypes and impacts of wind field direction on patterns of gene flow. As such, Landguth et al. is the first paper to combine empirical data, experimentation, and large-scale population-wide simulation modeling of adaptive evolution in spatially-complex landscapes. The second simulation paper, by Cushman and Landguth uses the same individual-based, spatially-explicit modeling approach to explore the interactions of heterogeneous environmental selection with speciation driven by hybrid incompatibility. Within-species hybrid incompatibility arises when combinations of alleles at more than one locus have low fitness but where possession of one of those alleles has little or no fitness consequence for the carriers. In this paper, Cushman and Landguth use simulation modeling to explore the effects of heterogeneous natural selection on the frequency, size and duration of reproductively isolated clusters of individuals in continuously distributed populations. They found that spatially heterogeneous selection produced clusters of reproductively isolated individuals that were much larger, longer lasting and spatially proximal. This pattern was strong across levels of gene flow and strength of selection, suggesting that even relatively weak selection acting in the context of strong gene flow may produce reproductively isolated clusters that are large and persistent, enabling incipient speciation in a continuous population without geographic isolation.

Another important topic in evolutionary theory and spatial genetics relates to the effects of past demographic events in species history on current patterns of genetic structure and differentiation. To address this issue, Cubry et al. argue that rare variants are important for drawing inference about past demographic events in a species' history, and specifically that singletons, which are variants for which genetic variation is carried by a unique chromosome in a sample, provide a particularly powerful lens to explore deep demographic history and its impacts on current population structure. They define the empirical distribution of singletons and then use computer simulations to evaluate the potential for the empirical distribution of singletons to provide a description of genetic diversity across geographic space. Using a Bayesian framework, they then show that this measure leads to accurate estimates of the geographic origin of range expansions and use this approach to estimate the origin of a cultivated plant species. Ultimately, this paper demonstrates that the empirical distribution of singletons is a useful measure to analyze results of sequencing projects based on large scale sampling of individuals across geographic space.

The three empirical case studies address two crop plants and one wild mammal species. In the first plant-based empirical example,  Egea et al. explore the genomics of garlic. They use high-throughput genotyping-by-sequencing approaches to assess genetic diversity and structure of a large garlic-germplasm bank, relate genotypes to agronomical history and develop a cost-effective method to manage genetic diversity in germplasm banks. They identified three main garlic-groups and demonstrated that DArTseq is a cost-effective method to analyze species with large and expected complex genomes, like garlic. In the second plant-based empirical study, Abebe et al. focused on detecting adaptive loci in barley. They also used a genotyping by sequencing approach on a diverse population of barley landraces and compared genomic structure to climatic data. Partitioning the variance between climate variables and geographic distance indicated that climate variables accounted for most of the explainable genetic variation, and analysis of the associated SNPs revealed putative candidate genes for plant adaptation. This study highlights the utility of landscape genomic approaches to detect the presence of putative adaptive loci among barley landraces. The final empirical case study Zero et al.) focuses on how the persistence of small populations is influenced by genetic structure and functional connectivity. The authors used two network-based approaches to understand the persistence of the northern Idaho ground squirrel (Urocitellus brunneus) and the southern Idaho ground squirrel (U. endemicus), two rare species. They found that population graph analyses revealed that local extinction rapidly reduced connectivity for the southern species, while connectivity for the northern species could be maintained following local extinction. Results from gravity models complemented those of population graph analyses and indicated that potential productivity and large-scale topographic features drove connectivity in the northern species. The paper is one of the very first examples of using scenario analysis in landscape genetics to inform conservation strategies of other species exhibiting patchy distributions.

The final paper in the Research Topic addresses spatial analysis itself. There are two components of landscape genomics: landscape analysis and genetic data. However, a large majority of work has focused primarily on the genetic data component of the field, and much less on methods, theory and best practices in spatial analysis. Obtaining reliable knowledge about the pattern-process relationships that govern population demographics and evolution in complex environments requires rigorous approaches to link genetic, genomic, and epigenetic data to environmental and spatial drivers. To begin to address this critical need, Leempoel et al. explore the use of Geographic Information Systems (GIS) in landscape genetics and landscape genomics. They note that GIS is a tool that is uniquely suited to overlaying genetic information with environmental data, which is the prerequisite to locate and analyze genetic boundaries of various plant and animal species or to study gene-environment associations (GEA). Their paper focuses on the power of free and open-source GIS approaches and provide essential information for their successful application in molecular ecology. The paper provides a useful introduction to the key concepts related to GIS and then presents an overview of open-source GIS-related software, file formats, major environmental databases. Then the authors focus on GIS applications in landscape genetics, such as sampling strategies for Next Generation Sequencing, data exploration and spatial statistics suited for the analysis of large genetic datasets, and provide suggestions to properly edit maps and to make them as comprehensive as possible.

The overall goal for this Research Topic was to produce a concentrated compilation of the current thinking, methods, and perspectives in the emerging field of landscape genomics. In that regard, the mixture of review papers, simulation modeling advances, empirical examples and methodological approaches, we hope, will serve the reader well as a broad, current overview of this field. We truly feel there are few subjects that can claim to have an equal degree of synergy and rapidity of development as landscape genomics. The collision of explosive advances in genomic data generation with powerful individual-based simulation modeling approaches, and their integration with experimental genetics studies, provides an incredibly powerful synergy that is transforming entire fields of genetics, ecology and conservation. We hope this Research Topic will serve in some small way to advance this exciting growth of knowledge.

Looking forward, we believe that advancing landscape genomics will depend on formally linking genomic datasets with modeling and experimentation (Cushman, 2014). The papers in this Research Topic provide some initial insight into the challenges of this integration and the current state of development in its several parts. Given that no single person has the expertise to effectively bring these components together, success in advancing our field will depend on collaborations across large multi-disciplinary groups. The broad range of topics and expertise represented in this Research Topic may be seen as the nucleus of such a cross-disciplinary effort at integration, but clearly there is a tremendous amount to be done and this initial step has, more than anything, revealed that. Experts genomic, epigenomic, and transcriptomic data must work with bioinformatics specialists to efficiently handle and analyze vast genomic datasets, and to effectively utilize high performance computing resources. Modelers and experimental geneticist must work collaboratively with the bioinformaticians and genomics experts to test hypotheses in controlled and replicated experiments and project the relationships identified into broad and complex landscaps in a rapidly changing world. Accelerating global change presents a tremendous threat to the biosphere and challenge to human civilization. Landscape genomics will provide extremely valuable tools and approaches to understand, predict and mitigate the negative effects of global change on biodiversity, but only if it progresses rapidly to integrate genomic data, spatial modeling and experimental genetics.

## Author contributions

All authors co-edited this Research Topic. SC wrote the first draft of this editorial, and the other co-authors provided edits and revisions.

### Conflict of interest statement

The authors declare that the research was conducted in the absence of any commercial or financial relationships that could be construed as a potential conflict of interest.
